# Validity Evidence for Interprofessional Performance Scale in Conference (IPSC) in Japan

**DOI:** 10.15694/mep.2019.000054.1

**Published:** 2019-03-14

**Authors:** Junji Haruta, Yu Yamamoto, Ryohei Goto, Takami Maeno

**Affiliations:** 1 University of Tsukuba

**Keywords:** Psychometrics, Assessment, Interprofessional, Decision-making, Effective, Healthcare professionals, Patient care

## Abstract

This article was migrated. The article was marked as recommended.

**Introduction:** Assessment of interprofessional learning (IPL) in practice is a key educational strategy. Especially, interprofessional collaboration in a conference is reasonable to assess interprofessional competency because we have many conferences for sharing information and coping with complex issues interprofessionally in practice. This study aimed to validity evidence supporting the IPL assessment scores, namely the Interprofessional Performance Scale in Conference (IPSC), and to evaluate its reliability.

**Methods:** As a content validity, to obtain a consensus concerning the IPSC, we held five workshops from June 2016 to January 2018. Covering a response process, healthcare professional participants (raters) assessed interprofessional performance using the developed IPSC by watching 3 different types of videos of case conferences, with 5 healthcare professionals participating in each. Rater agreement for the response process among six different raters was assessed using intraclass correlation coefficients (ICCs). Concerning internal structure, we examined the descriptive statistics and one-way analysis of variance as well as Generalizability (G) and Decision (D) studies. We explored the consequences of the IPSC through feedback sheet.

**Result:**The finalized IPSC assessment consisted of 12 items with 4-point Likert scales. ICC was 0.45 for the overall score. The association between IPSC and videos was significant while that of IPSC and professionals was not. The G and phi coefficients were 0.86 and 0.84, respectively. In descending order, the portion of the variance was professionals (15.7%) and the interaction of professionals and raters (11.5%) of the total variance. As D study, to reach a phi coefficient of 0.80, seven items and five raters were required. Through assessing interprofessional performance in case conferences, we clarified participants could reflect their own interprofessional competency as a consequence of the IPSC.

**Conclusion:**Findings from this study support using the IPSC as a tool to make consistent assessments of interprofessional performance in conferences.

## Introduction

In global health, the number of complex problems that healthcare practitioners must deal with from multiple perspectives is increasing ( Bateman *et al.*, 2001). To enhance the quality of care for complex healthcare needs, healthcare professionals should strive to improve healthcare team communication, efficiency and cohesiveness ( Gilbert, Yan and Hoffman, 2010). To this end, the last decade has seen increasing appreciation of the value of interprofessional learning (IPL) as a key educational strategy ( Gilbert, Yan and Hoffman, 2010). To date, however, no clear consensus on the best way to assess IPL has yet emerged ( Rogers *et al*., 2017).

Assessment plays an important role to drive learning ( Thistlethwaite, 2015) across summative and formative assessments ( Barr H, Gray R, Helme M, Low H, 2016). In addition, assessment is key to competency-based assessment ( Frenk *et al.*, 2010). Accordingly, we have previously discussed how to assess capability ( Gardner *et al.*, 2008; Brewer and Jones, 2013) and interprofessionalism ( Hall, Weaver and Grassau, 2013). In pre-registration, despite progress in the discussion on how to assess how IPL, few studies have investigated the assessment of post-registration or continuous IPL in practice. The assessment of IPL in practice is required for several reasons: to verify the ability to provide safe and effective practice; to meet the needs and expectations of patients, clients and communities, as well as families; and to ensure effective cooperation and interprofessional communication between healthcare professionals. For example, multi-professional trainees and staff who participate in interprofessional conferences can strengthen both their understanding of other professionals’ roles and their own interprofessional facilitation skills ( Sordahl *et al.*, 2018). These various benefits warrant further research into the assessment of interprofessional performance in a conference.

Moreover, assessment of IPL should be feasible, affordable and acceptable for all stakeholders, and able to acknowledge the sensitivity of interprofessional competency while encouraging learning ( Rogers *et al*., 2017). In this sense, interprofessional collaboration in a conference has been frequently implemented for sharing information and coping with complex issues. Therefore, we focused on interprofessional case conferences and developed the Interprofessional Performance Scale in Conference (IPSC) based on interprofessional competency frameworks in Japan ( Haruta *et al.*, 2018).

In this study, we aim to gather validity and reliability evidence supporting the use of the IPCS as a useful and acceptable approach to measure interprofessional performance based on an interprofessional competency framework for multidisciplinary healthcare practitioners.

## Methods

We conceptualized validity following Messick’s unified framework, gathering the following sources of validity evidence: content, response process, internal structure, and consequences of testing ( Downing, 2003; Downing and Yudkowsk, 2009).

*Content:* We developed the IPSC as an assessment tool in the form of a multidisciplinary consensus-based assessment scale to assess interprofessional performance in a case conference based on interprofessional competency in Japan, which includes 2 core domains and 4 peripheral domains ( Table 1).

**Table 1.** Interprofessional Competency Framework: competency domains and statements

Core domains	Statements
Patient-/Client-/Family-/Community-centered	Collaborative professionals can focus on important issues in which patients, clients, families, and communities are interested, and share goals in order to improve healthcare services for patients, clients, families, and communities.
Interprofessional Communication	Professionals respect other different professionals and share roles, knowledge, ideas, and values with each other for patients, clients, families, and communities.
**Peripheral domains**	**Statements**
Role Contribution	Professionals can understand mutual roles and utilize knowledge and skills with each other to conduct their roles properly.
Facilitation of Relationship	Professionals can build, maintain and grow relationships with different professionals. Professionals can also properly cope with conflicts among different professionals.
Reflection	Professionals can review concepts, performance, emotion, and values of their own professions, and powerfully understand their collaborative experiences with different professionals to improve collaboration.
Understanding of Others	Professionals can understand concepts, performance, emotion, and values of other professions to improve collaboration.

The core domains are: “Patient-/client-/family-/community-centered”, and “Interprofessional communication”, and the peripheral domains are “Role contribution”, “Facilitation of relationships”, “Reflection”, and “Understanding of others”. These domains almost exactly match the consensus for interprofessional outcomes in the following 6 domains, namely role understanding, interprofessional communication, interprofessional values, coordination and collaborative decision-making, reflexivity, and teamwork ( Rogers *et al.*, 2017).

A consensus method based on the subjective opinions of several experts is considered an appropriate way of developing a performance assessment ( Boursicot *et al.*, 2011; Sandberg *et al.*, 2015). To gather a wide range of multiprofessional opinions and to examine acceptance in practice, we held 5 public workshops focused on the assessment of IPL from 2016 to 2018 for multidisciplinary healthcare practitioners. Information on the workshops was disseminated through the homepage and mailing list of an academic organization. These workshops consisted of four sections: 1) introduction of the aim, schedule, and research participants; 2) a lecture about the interprofessional competency framework in Japan; 3) assessment using the prototype IPSC, with participants watching the simulated interprofessional case conference on site or by video; and 4) reflection on methods to assess interprofessional performance in conferences using the IPSC. In the setting of the simulated case conferences in these workshops, a physician in charge, ward nurse, pharmacist, physiotherapist, and medical social worker (MSW) participated in the case of a male patient who had been given a diagnosis of cerebral embolism caused by atrial fibrillation and who was admitted to a hospital. In the conference setting, he was advised after hospitalization for two weeks to transfer to a hospital with a recovery rehabilitation ward, despite wanting to be discharged home and receive outpatient care.

Prior to conducting this study, 2 researchers (physician, and specialist of psychological statistics) involved in the Interprofessional Competency Framework in Japan project, 3 healthcare professionals (medical assistant, occupational therapist, and speech therapist), and 2 of the authors (JH, YY) developed the 17 items of the IPSC as prototype 1. We then revised prototype 1 based on feedback from workshop participants of the Conference of the Japan Primary Care Association in June 2016 and a workshop in August 2016 to develop prototype 2. To obtain participants’ feedback, we created a feedback sheet with a four-point grading scale for assessment based on the overall quantification of content validity: 1=very acceptable; 2=mostly acceptable; 3=neutral; 4=not quite acceptable; and 5=not acceptable. If participants scored 3-5 points, they let us know the revised points through the feedback sheet. Second, to improve prototype 1, we revised the IPSC and asked for feedback repeatedly in October and December of 2016. Third, we likewise revised the IPSC and obtained feedback from the Japan Primary Care Association in 2017. Fourth, we finalized the Japanese version of the IPSC. Finally, we translated the IPSC into English, and obtained consensus for this translated version from workshop participants in the Asia Pacific Medical Education Conference in January 2018.

*Response process:* We developed 3 different types of video material. Video 1 showed the physician commanding the other professionals, especially contradicting the PT, and showed the other healthcare professionals eager to avoid displeasing the physician. Video 2 showed the MSW smoothly questioning each healthcare professional playing the role of moderator, and their collaboration in sharing constructive opinions. Video 3 showed the nurse insisting on her role contribution with regard to the other professionals, except for the physician. In particular, the MSW became nervous of the nurse and could not play an effective role.

Study participants as raters using purposive sampling were recruited from among participants of the previous workshops from 2016 to 2018, as described above. Eligible participants had over 5 years of professional experience. Each participant assessed one or two healthcare professionals in the 3 videos described above on the website. We allocated the participants such that 6 participants as raters could assess each professional participating in the simulated interprofessional case conference on the video. Using the data, we analyzed the intraclass correlation coefficient (ICC) to measure rater reliability ( Shrout and Fleiss, 1979). In addition, we assessed the percentage of variance among the raters using a generalizability study (G study) ( Bloch and Norman, 2012).

*Internal structure:* First, we examined the descriptive statistics (max, min, median, mean, and standard deviation) of IPSC scores, for all items in each of the 3 videos, and compared them. Second, to compare the impact of the 3 videos on the average of the 5 professionals’ total score of IPSC and the impact of the 5 professionals in each video on the average total score of the IPSC, a one-way analysis of variance (ANOVA) and Tukey’s multiple comparison test was used. *P* < 0.05 was considered to indicate a statistically significant difference for ANOVA. Third, we used the generalizability theory to analyze the reliability of scores from the observation data of the video using the IPSC ( Bloch and Norman, 2012). Generalizability theory was used to assess the internal structure of the IPSC in order to analyze the reliability of IPSC scores. All aspects included professionals (p) in the video, items (i) in the IPSC, and study participants as raters (r), as well as interaction terms. The phi coefficient was chosen over the G coefficient for all G studies, given that the IPSC represents a criterion-based measure. We also conducted decision studies (D studies) to project improvements in reliability for the varying number of raters and to determine the number of items that would need to be scored to assess interprofessional performance in a case conference.

*Consequences:*We explored the descriptive data through feedback sheets by using free descriptive answer text focused on how and what participants considered the assessment of interprofessional performance while watching the interprofessional conference video.

The study protocol was approved by the Institutional Review Board of the University of Tsukuba (No.1066).

## Results/Analysis

*Content:* A total of 75 healthcare professionals participated in revising the IPSC ( Table 2). The prototype 1 IPSC, which was developed based on the interprofessional competency framework in Japan, consisted of 17 items in all domains, namely 3"Patient-/client-/family-/community-centered" items, 4 “Interprofessional communication” items, 2 “Role contribution” items, 4 “Facilitation of relationships” items, 2 “Reflection” items, and 2 “Understanding of others” items. Acceptance rates by participants in the first two workshops were 14/18 (74%) and 10/15 (67%), respectively ( Table 2).

**Table 2.** Evidence of content validity

Prototype	Participants	Professionals	Gender	Number	Manner of watching the conference	Response rate	Acceptance rate
Prototype 1	The Conference of Japan Primary Care Association in June of 2016	Doctor	Male	16	on site	18/25(72%)	14/18(78%)
			Female	2			
		Pharmacist	Male	4			
			Female	3			
	The workshop in August of 2016	Doctor	Male	3	on site	15/26 (58%)	10/15 (67%)
		Dentist	Male	1			
		Nurse	Female	1			
		Dietician	Female	1			
		Psychologist	Female	1			
		PSW	Female	1			
		Unknown		7			
Prototype 2	Participants in the workshop in October 2016	Doctor	Male	1	Video	6/6 (100%)	5/6 (83%)
			Female	1			
		Pharmacist	Male	2			
		Nurse	Female	1			
		Psychologist	Female	1			
	Participants in the workshop in December of 2016	Doctor	Male	2	Video	4/4 (100%)	3/4 (75%)
		Medical assistant	Female	1			
		Pharmacist	Male	1			
Prototype 3	Participants in the Conference of Japan Primary Care Association in 2017	Doctor	Male	15	Video	19/19 (100%)	18/19 (95%）
			Female	1			
		Pharmacist	Male	2			
		Nurse	Female	1			
	Participants in the APMEC in 2018	Physician	Male	1	Video	5/6 (83%)	5/5 (100%)
			Female	5			
	Total		75				

After the first two workshops, the authors created prototype 2, which consisted of 15 items based on the feedback, namely 2 “Patient-/client-/family-/community-centered” items, 3 “Interprofessional communication” items, 2 “Role contribution” items, 4 “Facilitation of relationships” items, 1 “Reflection” item, and 2 “Understanding of others” items. Further, we added 1 “Coordination and collaborative decision-making” item, which was one of the consensus interprofessional outcome items in the 6 domains. Acceptance rates by participants in the middle two workshops were 5/6 (83%) and 3/4 (75%), respectively.

After the two intermediate workshops in October and December 2016, the authors created a final version consisting of 12 items based on the feedback received, namely 2 “Patient-/client-/family-/community-centered” items, 3 “Interprofessional communication” items, 2 “Role contribution” items, 2 “Facilitation of relationships” items, 2 “Understanding of others” items, and 1 “Coordination and collaborative decision-making” item. Further, they deleted the 1 “Reflection” item because raters were unable to assess reflection only by watching the care conference video of multidisciplinary healthcare professionals. The representations of items were slightly changed for ease of understanding. The acceptance rates by participants in the last two workshops were 18/19 (95%) and 5/5 (100%), respectively. The finalized IPSC included 12 items in 6 domains using 4-point Likert scales (4: Excellent, 1: Poor) and is shown in Table 2.

*Response process:*The 16 study participants (raters) who watched the 3 videos consisted of 6 doctors, 1 dentist, 1 nurse, 2 pharmacist, 4 therapists, 1 medical educator (non-healthcare professional), and 1 acupuncturist. As a result of the allocation of participants as raters, the healthcare professionals in each video were assessed by six different healthcare professionals (raters). The ICC was 0.45 for the overall score. ICCs for the other domain scores ranged from 0.13 to 0.53, which, except for the domain “patient centered care”, indicated fair or moderate agreement among the raters ( Table 3). Raters accounted for only a small proportion (1.5%) of the variance in G studies ( Table 4).

**Table 3.** Developed IPFS and rater agreement using ICC

	Observation Items	ICC	95% CI
Patient centered	Gets information on goals from patients/ users, families and multi-professions.	0.13	-0.11-0.4
	Focuses on important issues for patients/ users and families.	0.16	0.016-0.43
Interprofessional Communication	Listens firmly to information from other professions.	0.39	0.19-0.66
	Asks respectfully questions for the multi-professions.	0.45	0.24-0.70
	Shares information in easy-to understand terms that multi-professions can understand well.	0.32	0.12-0.59
Role Contribution	Conveys the patients’ and / or users’ situations and interventions from the viewpoint as self-profession.	0.3	0.10-0.58
	Conveys general knowledge and values of own profession.	0.21	0.04-0.48
Facilitation of Relationships	Asks participants in an easy to respond to way and promotes exchanging opinions	0.53	0.32-0.76
	Moderates the conference to proceed more smoothly	0.42	0.21-0.68
Understanding of Others	Works on making the best use of the roles of other professions	0.34	0.15-0.62
	Recognizes mutual values when faced with differences in values among multi-professions	0.32	0.13-0.60
Decision Making	Sets common goals and policies based on information of patients/ users and families.	0.41	0.21-0.68
Total		0.45	0.24-0.71

IPSC; Interprofessional Performance Scale in Conference

ICC; Intraclass correlation coefficients

**Table 4.** G study in IPSC

	Difference of freedom	Variance Components	Percent Variance %
Professionals (p)	14	0.2465	15.70%
Items (i)	11	0.0609	3.90%
Rater(r）	5	0.0231	1.50%
p*I	154	0	0%
p*r	70	0.1807	11.50%
i*r	55	0	0%
p*i*r	770	1.2005	76.30%

G study; Generalization study

IPSC; Interprofessional Performance Scale in Conference

*Internal structure:*In the IPSC, the possible scores range from 0 to 48 for total score and from 0 to 4 for each of the 5 criteria (including N/A=zero). The mean, maximum and minimum IPSC scores for each video were 27.7, 29.8 (MSW), and 25.2 (Doctor) in video 1; 38.5, 43.8 (Doctor), and 35.8 (Nurse and PT) in video 2; and 29.5, 35.2 (Doctor), 19.5 (Nurse) in video 3. Standard deviation was 10.3 (Doctor in video 1) as max and 2.8 (Pharmacist in video 2) ( Table 5).

**Table 5.** Descriptive statistics (Max, Min, Median, Mean, and SD) of IPSC scores

The video contents	Professionals	Max	Min	Median	Means	SD
Video 1：The physician commanded the other professionals, especially contradicted PT. The other healthcare professionals were eager to avoid displeasing the physician.	Physician	4	1	2	25.2	10.3
	Nurse	4	1	2	27.8	8.0
	Pharmacy	4	1	2	29.0	8.6
	PT	4	1	2	26.5	4.7
	MSW	4	1	3	29.8	5.0
	Group mean	4	1	2.2	27.7	7.3
Video 2: The MSW made smooth questions for every healthcare professional as the role of moderator and collaborator to share constructive opinions.	Physician	4	1	4	43.8	3.6
	Nurse	4	1	3	35.8	8.7
	Pharmacy	4	2	3	37.7	2.8
	PT	4	1	3	35.8	6.0
	MSW	4	2	4	39.3	8.7
	Group mean	4	1.4	3.4	38.5	6.0
Video 3: The nurse insisted on emphasizing her role contribution to the other professionals, except for the physician. Of note, the MSW got nervous of the nurse and cannot play its own role.	Physician	4	1	3	35.2	7.2
	Nurse	3	1	2	19.5	4.3
	Pharmacy	4	1	3	33.2	4.2
	PT	4	1	3	31.0	7.0
	MSW	4	1	2	28.8	5.7
	Group mean	3.8	1	2.6	29.5	5.7
	Total				31.9	6.0

SD; standard deviation

IPSC; Interprofessional Performance Scale in Conference

The F-test for the one-way ANOVA and Tukey’s multiple comparisons test between IPSC and videos showed a significant difference in mean (F=10.53, p <0.01); the difference of the average between video 1 and video 2 was 11.3 (CI 4.37-18.56, P=0.003); and the difference of the average between video 2 and video 3 was 9.3 (CI 2.24-16.63, P=0.01)). In contrast, the association between IPSC and professionals was not significant (F=0.438, P=0.779) ( Table 6, 7).

For the G study, we used data from 15 professionals (p) in the video, 12 items (i) in the IPSC, and study participants as raters (r) of the IPSC. The G and phi coefficients were 0.86 and 0.84, respectively ( Table 4). Professionals accounted for 15.7% of the total variance, constituting a large portion of variance. The second largest source of variance came from the interaction of professionals and raters (11.5%); that is, certain professionals were scored higher by one rater than by another. A large proportion of the residual error variance came from interactions between facets. Decision study (D study) results indicated that 7 items were required to reach a phi coefficient of 0.80 and 5 raters were required for a phi coefficient of 0.80.

**Table 6.** ANOVA using the 3 videos and 5 professionals’ total score of IPSC

Source of variance	SS	DF	MS	F	P
Inter Group of Video	371.91	2	185.96	10.53	0.00
Intra Group of Video	211.99	12	17.67		
Sum	583.90	14			
Inter Professionals in the Video	86.974	4	21.744	0.438	0.779
Intra Professionals in the Video	496.926	10	49.693		
Sum	583.900	14			

ANOVA; a one-way analysis of variance

IPSC; Interprofessional Performance Scale in Conference

**Table 7.** Tukey’s multiple comparison using the 3 videos on the average of the 5 professionals’ total score of IPSC

(I) Video		Difference of average	Standard Error	P	95% Confidence interval	
					Low	High
1	2	-11.5	2.66	0.003	-18.56	-4.37
	3	-2.1	2.66	0.709	-9.23	4.96
2	1	11.5	2.66	0.003	4.37	18.56
	3	9.3	2.66	0.011	2.24	16.43
3	1	2.1	2.66	0.709	-4.96	9.23
	2	-9.3	2.66	0.011	-16.43	-2.24

IPSC; Interprofessional Performance Scale in Conference

*Consequences:* We collected 137 descriptive data as sentences from all workshops. Many participants responded that they were able to compare their own interprofessional competency to the professionals’ performance in the video and that they reflected their own professional effect in interprofessional case conferences. Some participants reported that active listening and comprehension founded on opinions based on understanding of other professionals’ role were keys in interprofessional communication.

## Discussion

We gathered validity and reliability evidence supporting the use of IPSC, which functions not only for assessment but also as a reflection tool based on an interprofessional competency framework for multidisciplinary healthcare practitioners.

In real practice, IPSC may be mainly used for formative assessment because we tend to focus more on the learning process in post-registration or continuing professional development than our achievement ( Lobst *et al*., 2010). Thus, we need to use efficient and effective assessment strategies and tools for basing transition decisions on competence ( Carraccio *et al*., 2016). The findings of consequence using IPSC indicated that some healthcare professionals compared their own interprofessional performance to others, which might drive self-directed learning. Moreover, given the high workloads experienced by educational practices and clinical settings, we recommend the application of video assessment using IPSC. IPSC, which can be assessed on an interprofessional competency framework, may function seamlessly across the educational continuum from undergraduate to postgraduate and continuing professional development ( Lobst *et al.*, 2010). For instruments such as the iOSCE ( Simmons *et al.*, 2011; T Storjohann, A Herrick, KA Mitzel, LE Davis, C Thompson, 2017), which is an interprofessional OSCE, IPSC can be utilized from the undergraduate to postgraduate period and into continuing professional development.

We clarified that the team impact differs depending on the videos. In particular, the performance of doctors and nurses playing key roles, which might affect the development of scenarios in interprofessional care conferences, may have had substantial influence on the scoring given to the other professionals by individual professionals who participated in the interprofessional conference. For example, in video 1, in which the physician commanded the other professionals, the score of the other professionals was low. In video 3, in which the nurse insisted that her contribution be accepted by the other professionals, the score of not only the nurse but also the MSW was low. This might be associated with the general understanding that nurses and MSW need to collaborate to support a patient’s discharge. This finding indicates that interprofessional performance among professionals whose boundaries are ambiguous may be mutually influential. In contrast, physical therapists and pharmacists can make a significant contribution by actively sharing their own professional knowledge about patients. Based on these views, it is meaningful that physicians and nurses should reflect on whether through their attitude they have a negative effect on other professionals, and therapists and pharmacists should focus on sharing their own opinions based on their own knowledge.

In G theory, we can consider the role of social identity theory in the case of the second largest source of variance i.e. the interaction of professionals and raters. Social identity theory focuses on social structural factors ( Hogg *et al*., 2004; Ellemers and Haslam, 2012), such as medical hierarchy, that might affect an individual’s behavior, along with the actions expected of them by coded messages. Social identity attributes the cause of in-group favoritism to a psychological need for positive distinctiveness and describes situations in which in-group favoritism is likely to occur ( Giannakakis and Fritsche, 2011). For example, doctors tend to assess other doctors more highly than other healthcare professionals. In contrast, to protect the positive in-group image, in-group members are more critical of in-group deviants than deviants of an outgroup. These findings indicate that members of several healthcare professional groups should be conscious of bias, especially in assessing other professionals in their own group.

This study has several limitations. First, the number of participants was low, including raters and professionals of different types in the 3 videos. Similarly, the sample size used for both the G studies was small. In addition, some raters had previously participated in a workshop on the development of the IPSC and had a strong shared understanding of how to apply it. We did not explore validity evidence from this source because this was an exploratory study and there were no valid observational items or scales of interprofessional performance. Additional validity evidence is required to ensure that this observation scale can be used across real multidisciplinary practices. Comparison with other variables such as self-assessment is also warranted. Moreover, a larger and more heterogeneous sample of learners should be tested to confirm the generalizability of the results presented here.

## Conclusion

Findings from this study have meaningful implications for the assessment of interprofessional performance in conferences. The validity evidence presented here provides initial support for the use of the IPSC as an indicator of interprofessional performance in conferences. Additional studies which replicate the procedures in this study using larger and more heterogeneous samples are required. On the basis of these findings, we hope that utilization of the IPSC can become more widespread.

## Take Home Messages

• We developed and evaluated the Interprofessional Performance Scale in Conference (IPSC) using the Messick’s unified framework.
• Developed IPSC included 12 items in 6 domains and was validated by watching 3 different types of videos.
• ICC was 0.45 for the overall score, and the G and phi coefficients were 0.86 and 0.84.
• IPSC can become more widespread.

## Notes On Contributors

Junji Haruta is a family physician and researcher regarding primary care, medical education and interprofessional education. ORCID ID: https://orcid.org/0000-0003-4176-7665

Yu Yamamoto is a family physician with research regarding interprofessional education.

Ryohei Goto is a physical therapist with research regarding interprofessional education.

Takami Maeno is a family physician and researcher regarding interprofessional education.
