# Supplementary material for: Validity Evidence for Interprofessional Performance Scale in Conference (IPSC) in Japan
Source: MedEdPublish (2016). 2019 Mar 14;8:54. [Version 1] doi: 10.15694/mep.2019.000054.1 (PMC10712641; doi:10.15694/mep.2019.000054.1)
Supplement: Peer review history

# Reviewer report: Reviewer response for version 1

**DOI:** 10.21956/mep.19794.r29768

**Published:** 2019-03-22

**Authors:** Felix Silwimba

**Affiliations:** 1 University of Lusaka

This review has been migrated. The reviewer awarded 5 stars out of 5 The term interprofessional performance caught my attention and made me read through the article. I then realised this article is applicable to my situation as well. Even though my practice is in a middle-low income country where healthcare delivery is basic in nature. They are multiple interdisciplinary teams working to provide comprehensive healthcare to individuals and communities. Unfortunately, to larger extent they work towards undermining each other. Using this approach in the context of this environment could contribute significantly to harmonize multidisciplinary relations for better individual and community health outcomes. I recommend this study for a wider application across the globe.

Reviewer Expertise:

NA

No decision status is available

# Reviewer report: Reviewer response for version 1

**DOI:** 10.21956/mep.19794.r29766

**Published:** 2019-03-16

**Authors:** Balakrishnan Nair

**Affiliations:** 1 Centre for Medical Professional Development and University of Newcastle

This review has been migrated. The reviewer awarded 4 stars out of 5 Inter-professional collaboration is the only way to provide efficient patient centred care for majority of patients. However it is difficult to assess this in practice. So this study to validate a well known tool is an excellent initiative. Adult learn by reflection and the videos , they used, are an excellent way of doing this. The statistical analysis is well done and the article is well written

Reviewer Expertise:

NA

No decision status is available

# Reviewer report: Reviewer response for version 1

**DOI:** 10.21956/mep.19794.r29767

**Published:** 2019-03-14

**Authors:** Gerard Flaherty

**Affiliations:** 1 National University of Ireland Galway

This review has been migrated. The reviewer awarded 3 stars out of 5 The authors provide a psychometric report on the development and validation of an original assessment tool which they believe could have applications in the assessment of interprofessional learning in practice. The study methodology is rigorous and the design of the IPSC scale (termed “IPCS” in the final paragraph of the introduction) is based on a sound theoretical framework. I wonder to what extent the finalised instrument serves its original purpose, to assess interprofessional competency. I agree with its proposed use as a “reflection tool”. It may prove valuable in allowing multidisciplinary learners to self-assess their own learning as attendees at case conferences (incorrectly termed “care conference” in paragraph 2 of Page 6) and in this context, it could facilitate group debriefing sessions involving learners from diverse disciplines. I could see it being used as an assessment tool in a double video station in an OSCE, for example. The student would view a clip from a simulated case conference and then discuss it with an examiner at the succeeding station. The authors should expand on the possible utility of the IPSC in the discussion. In relation to the content validation, it is of note that 64% of the participants were male. The authors should comment on this gender imbalance and whether it would influence the validity of the ratings. Finally, some minor typos/corrections which the authors can easily address: (1) Abstract, Introduction: Change “validity” to “validate”; (2) No need to underline the domains in column 1 of Table 1; (3) Page 6, paragraph 2: Change Table 2 to Table 3; (4) In the caption for Table 3, change “IPFS” to “IPSC”; (5) I found Table 5 and the preceding paragraph very confusing. Why are the mean and median so different? It was on a 4-point Likert scale so should the mean also not lie between 1 and 4, or have I misinterpreted the table?; (6) Provide footnotes for the 5 acronym headers (SS….P) in Table 6; (7) It would be interesting to view a summary, perhaps in a single table, of the qualitative data from the workshops referred to briefly under “Consequences” on Page 9. Overall, this was a well conducted study which describes the development of a potentially useful assessment/reflective method in the setting of multidisciplinary case conferences, a very rich but under-utilised source of interprofessional learning.

Reviewer Expertise:

NA

No decision status is available
